# Post-bronchoscopy fatal endobronchial hemorrhage in a woman with bronchopulmonary mucormycosis: a case report

**DOI:** 10.1186/1752-1947-4-398

**Published:** 2010-12-09

**Authors:** Paola Di Carlo, Daniela Cabibi, Anton Maria La Rocca, Dario De Luca, Francesco La Licata, Ennio Sacco

**Affiliations:** 1 Infectious Diseases Section, Department of Health Promotion Sciences, University of Palermo, Palermo, Italy; 2 Department of Human Pathology, University of Palermo, Palermo, Italy; 3 Rehabilitation Unit, G.F. Ingrassia Hospital, Palermo, Italy; 4 Bronchology Unit, V. Cervello Hospital, Palermo, Italy

## Abstract

**Introduction:**

During infection, Mucorales fungi invade major blood vessels, leading to extensive necrosis, and in cases of extensive pulmonary disease, bleeding into the lungs may occur.

**Case presentation:**

We report an unexpected event of post-bronchoscopy fatal endobronchial hemorrhage in a 62-year-old HIV-negative Italian woman with well controlled diabetes mellitus who presented with diffuse cavitated pulmonary lesions. Fiberoptic bronchoscopy revealed bilateral obstruction of the segmental bronchi. Fatal massive bleeding occurred after standard biopsy procedures. Histologic examination showed that the hyphae were more deeply colored by hematoxylin-eosin (H&E) than by other stains for fungi. Culture and autopsy confirmed bronchopulmonary mucormycosis.

**Conclusion:**

Infection by Mucorales fungi should be considered in the diabetes population regardless of the degree of metabolic control. In these patients, particular caution should be taken during bronchoscopic procedures because of the greater friability of the fungal lesions.

## Introduction

"Zygomycosis" refers to infections caused by a class of fungi called Zygomycetes, which includes the genera *Rhizopus*, *Absidia*, and *Rhizomucor*. They had previously been assigned to the genus *Mucor *and were considered responsible for the disease known as "mucormycosis" [1,2]. These fungi are ubiquitous in nature and are common inhabitants of decomposing matter. They can cause serious and rapidly fatal infections, particularly in individuals with compromised immune systems, such as those with poorly controlled diabetes with ketoacidosis [1-4].

The fungi invade major blood vessels, leading to extensive necrosis, and in extensive pulmonary disease, bleeding into the lungs may occur. In patients with diabetes mellitus, pulmonary mucormycosis may develop, with a less fulminant disease course but with atypical presentation of a solitary nodule [5].

Biopsy (surgical or transbronchial) of abnormal tissue retrieved by bronchoscopic aspiration or bronchoalveolar lavage (BAL) via a bronchoscope and microbiologic evaluation are the most efficient methods for detecting endobronchial Mucor [3-5]. We report a rare case of diffuse pulmonary mucormycosis in a patient with well-controlled type 2 diabetes who had a fatal pulmonary hemorrhage during a fiberoptic bronchoscopy procedure.

## Case presentation

**Figure 1:**
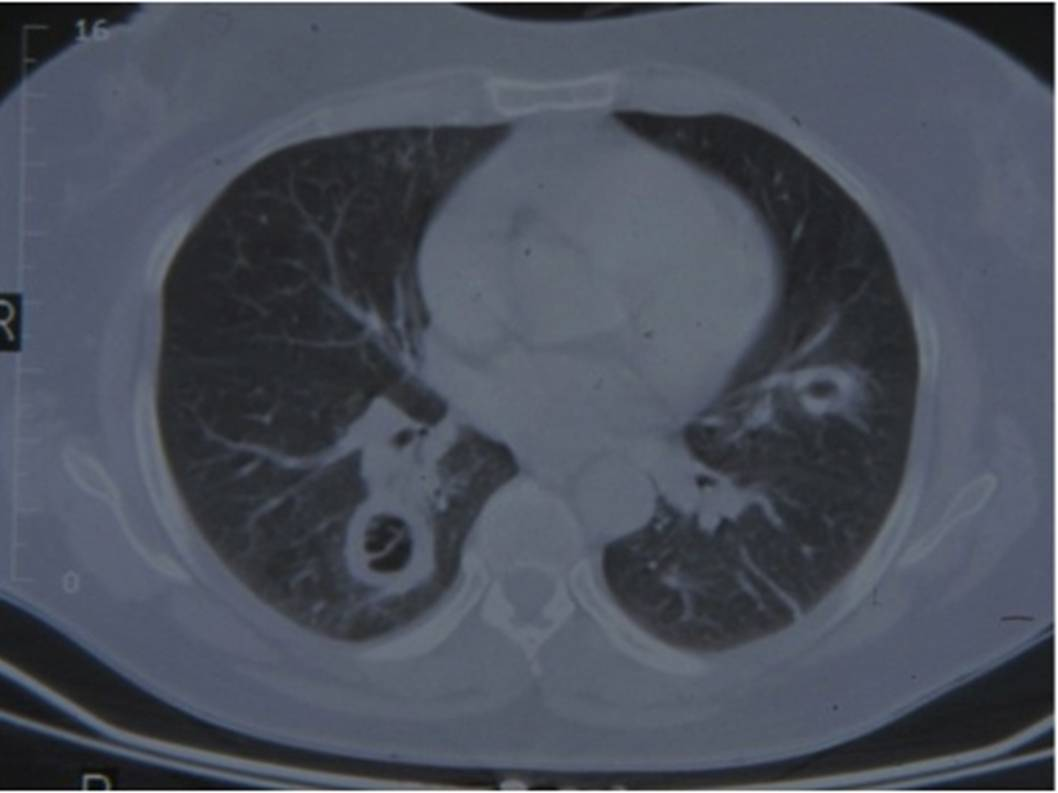
**Transverse computed tomography scan shows circular masses in pulmonary fields**.

A 62-year-old woman of Italian origin and nationality with a history of fever and a persistent cough for three weeks was admitted to our hospital for a scheduled fiberoptic bronchoscopy (FB) to assess the nature of a diffuse pulmonary lesion revealed by computed tomography (CT) chest scan (Figure 1).

Multiple cavitated lesions in the lungs were diagnosed. Some contained air and had a hyperdense capsule in the bronchi, consistent with bronchiectasis, whereas others resembled heteroplastic cavitary lesions (Figure 1). The patient had no significant clinical history except for type 2 diabetes mellitus (DM), controlled with oral anti-diabetic treatment and a suitable diet. In the three years of follow-up, her hemoglobin A_1c _remained below 7%.

At the time of admission to hospital, the patient's white blood cell count was 10 × 10^9^/L with 50% neutrophils; hemoglobin and blood glucose were 13.0 g and 260 mg/100 ml, respectively. No coagulation alterations were observed, and the thrombocyte count was 200.000 cells/ml. A human immunodeficiency virus (HIV) test performed on admission was negative.

**Figure 2:**
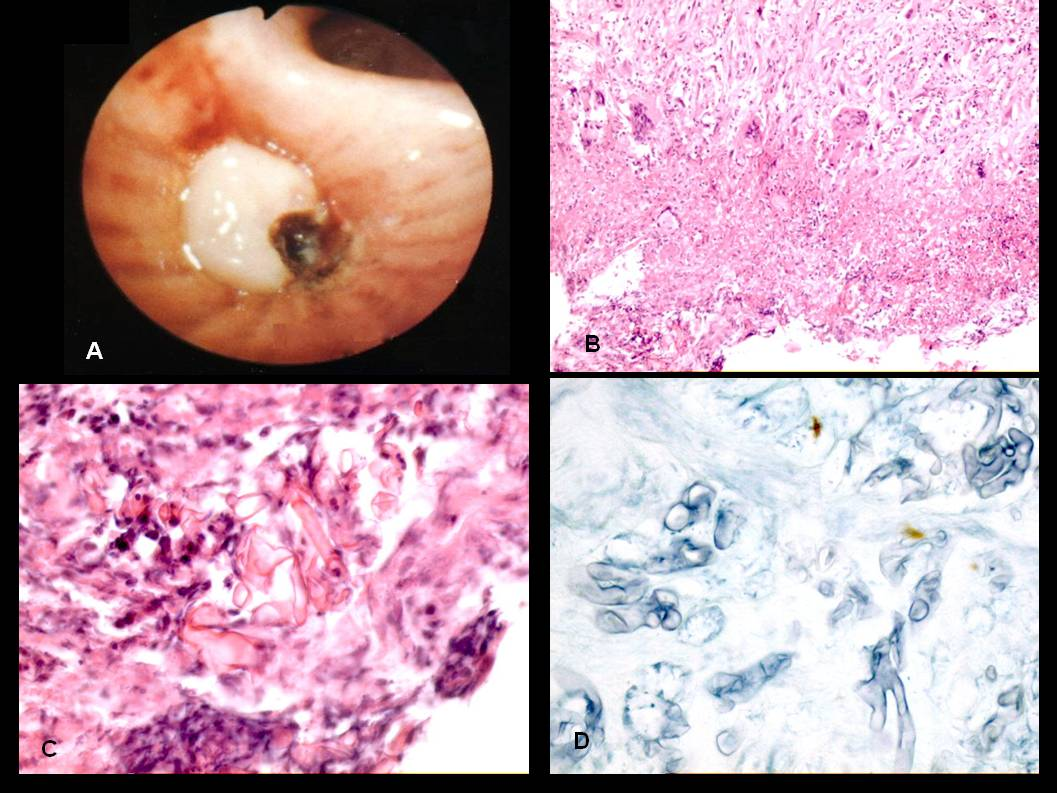
**Bronchoscopic image shows muconecrotic tissue**. **(a) **On histologic examination, necrotic tissue reveals large areas of necrosis with peripheral granulomatous reaction and several multinucleated cells; in the necrotic areas, numerous broad, very rarely septate, haphazardly branched hyphae were evident; the hyphae were deeply stained by H&E stain, more so than by PAS staining **(b-d)**. H&E (b) ×100; (c) ×400; PAS (d) ×400.

The bronchoscopy examination revealed a mucous and necrotic plug completely occluding the lingular bronchus and the apical segment of both lower-lobe bronchi (B6) (Figure 2a). Grasping forceps were used to remove mucous plugs for cytohistologic analysis, and other samples were obtained by bronchial aspiration. At the end of the sampling procedures, performed by a team of bronchologists with more than 20 years of experience in these techniques, the patient had a massive hemorrhage followed by cardiorespiratory arrest and was transferred to the Intensive Care Unit (ICU) in a state of coma.

Histologic examination showed large areas of necrosis in the bronchial mucosa, with peripheral granulomatous reaction and several multinucleated cells (Figure 2b). In the necrotic areas, numerous broad, very rarely septate, haphazardly branched hyphae were evident. Significantly, the hyphae were more deeply stained by hematoxylin-eosin (H&E) than by other special stains for fungi, such as periodic acid Schiff (PAS) (Figure 2c, d) and Gomori methenamine silver stain (GMS).

Morphologic aspects and stain affinity suggested mucormycosis. This hypothesis was confirmed by the results of the bronchial aspirate culture (histologic sample), which showed filamentous mycetes belonging to the class Zygomycetes. Consequently, the patient was treated with 5 mg of liposomal amphotericin B per pound of body weight.

No evidence of disseminated infection was found, and analysis of cerebrospinal fluid did not reveal cerebral mycoses. The patient died 20 days after being admitted to the ICU. Autopsy confirmed bronchopulmonary mucormycosis.

## Discussion

Mucormycosis can be radiologically misdiagnosed as active tuberculosis, chronic necrotizing aspergillosis, coccidioidomycosis, or bronchiectasis. These have all been reported in the diabetes population and may be associated with massive or recurrent hemoptysis [6-8].

Therefore, fiberoptic bronchoscopy with biopsy or bronchial aspirate or both are needed to confirm a suspected diagnosis and to start an appropriate therapy.

Previous reports of rhinocerebral or pulmonary mucormycosis in HIV-negative diabetes patients involved subjects with poorly controlled diabetes: acidosis and hyperglycemia provide an excellent environment for the fungus to grow [9].

In our patient's case, no evidence of severe or persistent hyperglycemia was noted. Her general condition and absence of coagulation alterations indicated that a bronchoscopy examination could be carried out. However, the fatal hemorrhagic event occurred during standard bronchoscopic procedures performed to obtain specimens for histologic and microbiologic assessment. Autopsy findings confirmed that endobronchial mucous and necrotic plugs seen during the bronchoscopic procedure were related to pulmonary vascular invasion by mucoraceous hyphae.

Fiberoptic bronchoscopic examination is a useful procedure for identifying bronchial obstructions and endoluminal lesions, as well as for assessing the tracheobronchial tree beyond stenoses. Moreover, the procedure makes it possible to restore normal airflow in airless areas around the blockage (possible atelectasis or subatelectasis). Al Majed [10] reported the removal of a mucormycosis lesion through a rigid bronchoscope.

The risks associated with bronchoscopy procedures are well known. However, our case study suggests that, in the absence of a clear or well-defined diagnosis, particular caution should be exercised when conducting an endoscopic examination in diabetes patients with suspected pulmonary mucormycosis. Similar to other cases of this group of fungi, angioinvasion, thrombosis, and necrotic lesions are the hallmark features. Moreover, diabetes patients have endothelial dysfunction, increased arterial stiffness, or decreased arterial distensibility [11]. Therefore, any sampling procedure such as aspiration may trigger vessel rupture, with massive bronchial hemorrhage.

In these cases, BAL could be advocated as a less invasive technique. Moreover, non-invasive techniques, such as virtual bronchoscopy, have been found to be useful for assessing lesion friability in cavitated disease [12].

It has been suggested that an air-crescent sign on a chest radiograph is an important sign of potentially fatal hemoptysis [13]. Moreover, lesion changes and progression should indicate the need to start early antifungal and surgical therapy [4]. However, in our case, none of these signs was observed.

Starting treatment early seems to be the significant factor in reducing mortality associated with this disseminated pulmonary disease. Most patients with mucormycosis have been treated with lipid preparations of amphotericin (predominantly liposomal) with few reactions or adverse events [3]. For years, amphotericin B has been the drug of choice for these highly aggressive infections. Recently, patients who were unresponsive to monotherapy with liposomal amphotericin B responded favorably to the addition of echinocandin caspofungin acetate [14]. Newly introduced, second-generation triazoles include voriconazole, which is not active against the Zygomycetes, and posaconazole, which has been demonstrated to be active *in vitro*, in animal models, and in case reports [15].

Other mechanisms to prevent or limit this fatal complication are unclear.

## Conclusion

Two interesting findings emerge from this case study. First, that mucormycosis should be considered in all diabetes patients regardless of degree of metabolic control.

Second, that fungal lesions may be more friable in these subjects, who might be at greater risk of complications associated with broncoscopy procedures.

## Abbreviations

GMS: Gomori methenamine silver stain; H&E: hematoxylin-eosin; PAS: periodic acid Schiff.

## Competing interests

The authors declare that they have no competing interests.

## Consent

Written informed consent was obtained from the patient's family for publication of this case report and accompanying images. A copy of the written consent is available for review by the Editor-in-Chief of this journal.

## Authors' contributions

PD, DC, and DD, participated in the conception of the idea, review of the literature, writing of the manuscript, and interpretation of histologic assays. FL collected and interpreted data. AML and ES wrote the pathologic section and reviewed the manuscript. All authors have read and approved the final manuscript.
